# Effects of the C-Terminal Truncation in NS1 Protein of the 2009 Pandemic H1N1 Influenza Virus on Host Gene Expression

**DOI:** 10.1371/journal.pone.0026175

**Published:** 2011-10-12

**Authors:** Jiagang Tu, Jing Guo, Anding Zhang, Wenting Zhang, Zongzheng Zhao, Hongbo Zhou, Cheng Liu, Huanchun Chen, Meilin Jin

**Affiliations:** Unit of Animal Infectious Diseases, National Key Laboratory of Agricultural Microbiology, College of Veterinary Medicine, Huazhong Agricultural University, Wuhan, People's Republic of China; Johns Hopkins University - Bloomberg School of Public Health, United States of America

## Abstract

The 2009 pandemic H1N1 influenza virus encodes an NS1 protein with 11 amino acids (aa) truncation at the C-terminus. The C-terminal tail of influenza virus NS1 protein constitutes a nucleolar localization signal (NoLS) and is the binding domain of the cellular pre-mRNA processing protein, poly(A)-binding protein II (PABII). Here, our studies showed that the C-terminal-truncated NS1 of the 2009 pandemic virus was inefficient at blocking host gene expression, extension of the truncated NS1 to its full length increased the inhibition of host gene expression. Mechanistically, this increased inhibition of host gene expression by the full-length NS1 was not associated with nucleolar localization, but was due to the restoration of NS1's binding capacity to PABII. Furthermore, *in vitro* and *in vivo* characterization of two recombinant viruses encoding either the C-terminal 11-aa truncated or full-length NS1 of the 2009 pandemic virus showed that the C-terminal 11-aa truncation in NS1 did not significantly alter virus replication, but increased virus pathogenicity in mice.

## Introduction

A novel swine-origin H1N1 influenza virus emerged in Mexico in April 2009 [1]–[2] and had spread globally prompting the World Health Organization (WHO) to raise this global pandemic to phase 6. Although it caused mild disease in the majority of people, the 2009 pandemic virus was more pathogenic and induced higher amounts of pro-inflammatory cytokines and chemokines in mammalian models than human seasonal H1N1 influenza viruses [3]. Interestingly, the majority of human seasonal H1N1 influenza viruses encoded a 230-aa NS1, while the 2009 pandemic H1N1 influenza virus encoded a 219-aa NS1 with an 11-aa truncation at the C-terminus [4].

The NS1 of influenza virus is a multifunctional protein that performs a number of activities, which may contribute to efficient virus replication and virulence during infection [5]. NS1 protein inhibits host antiviral gene expression by both pre-transcriptional and post-transcriptional processes. The pre-transcriptional inhibition occurs by preventing dsRNA- and virus-mediated activation of the transcription factors (IRF-3, NF-κB and c-Jun/ATF-2), which are essential for IFN-β production [6]–[8], while the post-transcriptional inhibition is involved in limiting cellular mRNA maturation and nucleo-cytoplasmic transport through interaction of NS1 protein with the 30 KD cleavage and polyadenylation specificity factor (CPSF30) or PABII [9]–[10]. In addition, NS1 can block the function of two antiviral proteins including 2′-5′-oligoadenylate synthetase (OAS) [11] and serine/threonine protein kinase R (PKR) [12], stimulate phosphoinositide 3-kinase signaling (PI3K) by binding to p85β and/or CrkL [13]–[16], and limit host antiviral gene translation by binding to eIF4GI [17].

The influenza virus NS1 contains two nuclear localization signals (NLS), which mediate the active nuclear import of NS1 via binding to celluar impotin-α [5]. The NLS1 is located in the N-terminal RNA-binding domain, while the NLS2 is located at the C-terminus. Certain critical amino acids (219K, 220R, 224R and 229K) of the C-terminal NLS2 constitute an NoLS [18]. The C-terminal 11-aa truncation (220-230) in NS1 of the 2009 pandemic virus might have lost the NoLS and affect intracellular localization of the NS1 protein.

In most eukaryotic cells, CPSF, cleavage stimulation factor (CstF), cleavage factors I and II (CFI and II) and poly(A) polymerase (PAP) are usually required for the cleavage of host pre-mRNAs and adding a short (∼10 nucleotide) poly(A) tail. Subsequent elongation of this short poly(A) tail requires PABII, CPSF and PAP, which facilitate rapid, processive poly(A) addition [9]–[10]. In addition, PABII also plays a role in nuclear export of host mRNA. Influenza virus NS1 can bind to CPSF30 and PABII and thus inhibits host pre-mRNA processing. The C-terminal amino acids 223-230 of NS1 constitute the PABII binding domain [5]. Whether the C-terminal 11-aa truncated NS1 of the 2009 pandemic virus has lost PABII binding capacity needed to be determined.

Here, we assessed the effects of the C-terminal 11-aa truncation in NS1 of the 2009 pandemic virus on host gene expression, nucleolar localization, PABII-binding capacity as well as virus replication and pathogenicity. In this study, we found that the C-terminal 11-aa truncated NS1 was inefficient at blocking host gene expression, failed to localized in nucleolus and lost the PABII-binding capacity. Extension of the C-terminal truncated NS1 to its full length increased the inhibition of host gene expression, restored PABII-binding capacity, but still did not localize in nucleolus. Moreover, the NS1 C-terminal 11-aa truncation did not significantly alter virus replication *in vitro* or *in vivo*, but enhanced virus virulence in mice.

## Materials and Methods

### Cells and plasmids

Human embryonic kidney (293T) cells, human lung epithelial (A549) cells, porcine kidney (PK-15) cells and Madin-Darby canine kidney (MDCK) cells were obtained from the American Type Culture Collection (ATCC) and maintained in Dulbecco's minimal essential medium (DMEM) (Invitrogen) with 10% fetal bovine serum (GIBCO, Auckland, NZ). The pHW-Mex NS plasmid was constructed by synthesizing the sequence of NS gene (accession number: GQ379816.1) from the 2009 pandemic virus A/Mexico/4486/2009 H1N1 (Mex) and cloning it into the pHW2000 plasmid as described previously [19]. A QuikChange XL site-directed mutagenesis kit (Stratagene, La Jolla, CA) was used to generate a mutant plasmid pHW-Mex mNS, in which the stop codon at aa position 220 of NS1 was changed from TGA to CGA. This alteration extended the NS1 C-terminus to the natural stop codon at aa position 230 without affecting the NS2 open reading frame (ORF). The plasmids pcDNA-Mex NS1/219, pcDNA-Mex NS1/230, pEGFP-Mex NS1/219, pEGFP-Mex NS1/230, pCMV-Mex NS1/219 and pCMV-Mex NS1/230 were constructed by ligating the PCR-amplified cDNAs containing different-length NS1 ORFs from the Mex strain into the plasmids pcDNA3.1, pEGFP-N1 or pCMV-Tag2B. The plasmids pcDNA-GST and pMYC-PABII were constructed by ligating the PCR-amplified cDNA of GST or PABII into the plasmids pcDNA3.1 or p-C-MYC.

### Virus generation by reverse genetics

An 8-plasmid reverse genetics system was used to generate recombinant viruses as described previously with some modification [19]. 293T cells were co-transfected with the seven plasmids (pHW-PR8 PB2, pHW-PR8 PB1, pHW-PR8 PA, pHW-PR8 HA, pHW-PR8 NP, pHW-PR8 NA and pHW-PR8 M) encoding each gene of virus A/PR/8/34 H1N1 (PR8) and plasmid pHW-Mex NS or pHW-Mex mNS. Supernatants were collected at 60 h post-transfection and injected into 10-day-old embryonated specific-pathogen-free (SPF) chicken eggs. Allantoic fluids were collected at 72 h post-infection (p.i.) and then subjected to a hemagglutination assay (HA). The generated recombinant viruses were named rPR8-Mex NS1/219 and rPR8-Mex NS1/230.

### Effects of the different-length NS1 protein on host gene expression

A549 cells in 12-well plates were co-transfected with 0.05 µg of Renilla luciferase plasmid pRL-TK (Promega, WI) and 0.5 µg of the expression plasmid pcDNA-GST, pcDNA-Mex NS1/219 or pcDNA-Mex NS1/230 using FuGENE HD (Roche, WI). At 24 h post-transfection, Renilla luciferase expression was measured according to the manufacturer's instructions (Promega, WI), and the total amounts of 219-aa NS1 and 230-aa NS1 protein expression were measured by Western blotting using rabbit anti-serum against a fusion protein of GST and Mex NS1/219, monoclonal antibody against tubulin was used to detect tubulin expression [20].

To test the effect of different-length NS1 protein on host antiviral gene IFN-β expression in infected cells, confluent A549 cells in 6-well plates were infected with recombinant virus rPR8-Mex NS1/219 or rPR8-Mex NS1/230 at a multiplicity of infection (MOI) of 3 TCID_50_ per cell, or equal volum of DMEM as control. At 6, 12 and 24 h p.i., the total RNA was extracted with RNeasy mini kit (Qiagen, CA) from infected cells or mock cells and subjected to DNase treatment (Qiagen, CA). Host antiviral gene IFN-β mRNA and viral M gene mRNA production were detected by quantitative real-time PCR (qRT-PCR) as described previously [21].

### Intracellular localization of the different-length NS1 protein in transfected and infected cells

A549 cells in 6-well plates were transfected with 2.0 µg of plasmid pEGFP-Mex NS1/219 or pEGFP-Mex NS1/230 using FuGENE HD (Roche, WI). At 24 h post-transfection, cells were imaged by an inverted fluorescence microscope (Olympus IX70, Japan).

To study intracellular localization of different-length NS1 protein in infected cells, A549 cells were infected with viruses rPR8-Mex NS1/219 or rPR8-Mex NS1/230 at an MOI of 3 TCID_50_ per cell. At 8 h p.i., cells were fixed with 4% paraformaldehyde, permeabilized with 0.5% Triton X-100 and incubated for one hour with 2% bovine serum albumin. NS1-specific rabbit anti-serum incubation was performed overnight at 4°C followed by FITC-labelled goat anti-rabbit IgG and 4′,6-diamidino-2-phenylindole (DAPI). Cells were imaged on a LSM510 Meta confocal laser scanning microscope.

### Interaction of the different-length NS1 protein with PAB II

293T cells in 6-well plates were co-transfected with 2.0 µg of the MYC-tagged vector pMYC-PABII and 2.0 µg of the FLAG-tagged vector pCMV-tag2B (empty vector), pCMV-Mex NS1/219 or pCMV-Mex NS1/230 using FuGENE HD (Roche, WI). Cells were lysed in 50 mM Tris-HCl (pH 7.8), 500 mM NaCl, 5 mM EDTA and 0.5% NP-40 supplemented with a protease inhibitor cocktail (Amresco) and subjected to anti-FLAG affinity chromatography at 48 h post-transfection. After being washed extensively, the precipitated proteins were dissociated from the resin using elution buffer and analyzed by SDS-PAGE followed by Western blot with anti-FLAG (Abcam) and anti-MYC (Sigma) antibodies. The proteins were visualized using a SuperSignal West Pico (Pierce) and detected with a MF-ChemiBIS system (DNR).

### *In vitro* characterization of the recombinant viruses encoding different-length NS1 protein

Growth kinetics of the two recombinant viruses rPR8-Mex NS1/219 and rPR8-Mex NS1/230 was determined by inoculating A549 or PK-15 cells at an MOI of 0.001 TCID_50_ per cell. One hour after inoculation, cells were washed twice with PBS and fresh medium containing 0.1 µg/ml tosylsulfony phenylalanyl chloromethyl ketone (TPCK)-trypsin (Sigma) was added. Supernatants were sampled at 12, 24, 36 and 48 h p.i., and virus titers were determined by calculating the log_10_TCID_50_/ml in MDCK cells.

Virus plaque assay of the two recombinant viruses rPR8-Mex NS1/219 and rPR8-Mex NS1/230 was performed by inoculating confluent MDCK cells in 6-well plates with appropriate dilutions of viruses in DMEM for 1 h. Cells were washed twice with PBS and then overlaid with DMEM (without phenol red, HyClone)-0.8% agarose mixture containing 1% bovine serum albumin and 1 µg/ml TPCK-trypsin. After incubated at 37°C for 4 days, a second agar overlay containing 1∶10,000 neutral red was added [17].

### Mouse experiments

Animal studies were performed according to protocols approved by the Hubei Provincial Animal Care and Use Committee (approval number: SYXK 2010-0029).

The fifty percent mouse lethal dose (MLD_50_) titers were determined by inoculating groups of four 5-week-old female BALB/c mice intranasally (i.n.) with 50 µl of serial 10-fold dilutions of the virus rPR8-Mex NS1/219 or rPR8-Mex NS1/230. Titers were expressed as the log_10_TCID_50_ required to achieve 1 MLD_50_.

In a separate experiment, groups of mice were infected i.n. with 50 µl of 10^2^ TCID_50_ of the recombinant virus rPR8-Mex NS1/219 or rPR8-Mex NS1/230. Body weights of five mice in each group were measured for either 14 days p.i. or until the loss of 30% of their body weight, at which point they were euthanized. The survival percentage of four mice in each group was monitored until 14 days p.i.. At days 3 and 6 p.i., six mice in each group were sacrificed, and the lungs were removed; three lungs were fixed with formalin, embedded in paraffin and stained with hematoxylin and eosin (HE), while the other three lungs were homogenized in 1 ml PBS to determine virus titers (log_10_TCID_50_/ml) in MDCK cells. For microarray analysis of mouse lungs infected with recombinant virus rPR8-Mex NS1/219 or rPR8-Mex NS1/230, at days 3 p.i., the lungs of three mice in each group were removed and subjected to microarray analysis (Affymetrix) as described previously [22]. The results of microarray data were confirmed by qRT-PCR as described previously [22].

## Results

### The C-terminal truncated NS1 of the 2009 pandemic virus was unable to block host gene expression

**Figure 1:**
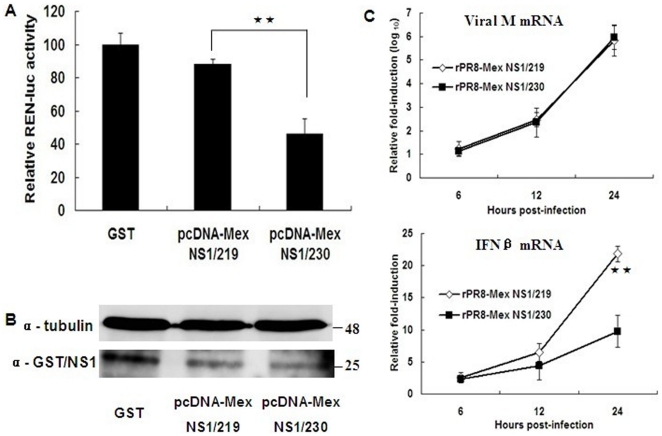
Inhibition of host gene expression by different-length NS1 protein. (A) A549 cells were co-transfected with Renilla luciferase plasmid pRL-TK and expression plasmid pcDNA-GST, pcDNA-Mex NS1/219 or pcDNA-Mex NS1/230. Renilla luciferase expression was measured at 24 h post-transfection. The results showed the means and standard deviations of triplicate values (normalized to GST). The statistical analysis was performed using Student's t test (★★, *p*<0.01; ★, *p*<0.05). (B) The 219-aa NS1 and 230-aa NS1 protein expression at 24 h post-transfection in transfected A549 cells was detected by Western bloting using rabbit anti-serum against a fusion protein of GST and Mex NS1/219, tubulin was used as control. (C) A549 cells were infected at an MOI of 3 TCID_50_ per cell. At 6, 12 and 24 h p.i., host antiviral gene IFN-β mRNA and viral M gene mRNA production was deteced by qRT-PCR. The statistical analysis was performed using Student's t test (★★, *p*<0.01; ★, *p*<0.05).

The NS1 of the classical swine H1N1 influenza virus was maintained at a length of 230 aa until the mid-1960s, a stop codon emerged at position 220 and caused an 11-aa truncation at the C-terminus [23]. This 219-aa NS1 has subsequently been predominant in the classical swine H1N1 influenza viruses and emerged in the 2009 pandemic H1N1 influenza virus. To test whether the C-terminal 11-aa truncation in NS1 of the 2009 pandemic virus has any effects on host gene expression, we co-transfected Renilla luciferase plasmid pRL-TK with expression plasmid pcDNA-GST, pcDNA-Mex NS1/219 or pcDNA-Mex NS1/230. At 24 h post-transfection, Renilla luciferase expression and the total amounts of NS1 protein expression were determined. As shown in Figure 1, similar amounts of 219-aa NS1 and 230-aa NS1 were expressed in the transfected cells (Figure 1B), while the 230-aa NS1 significantly increased the inhibition of host gene expression than did the 219-aa NS (Figure 1A).

We also meassured the antiviral gene IFN-β mRNA production in A549 cells infected with recombinant virus rPR8-Mex NS1/219 or rPR8-Mex NS1/230 by qRT-PCR. Viral M gene mRNA production was similar at each time point, while the antiviral gene IFN-β mRNA production was higher when cells were infected with virus rPR8-Mex NS1/219 than with virus rPR8-Mex NS1/230 (Figure 1C).

In addition, the effect on host gene expression *in vivo* of the C-terminal 11-aa truncation in NS1 of the 2009 pandemic virus was evaluated. Microarray analysis of infected mouse lungs at days 3 p.i. showed that the recombinant virus rPR8-Mex NS1/219 induced higher levels of pro-inflammatory cytokines and chemokines in mice lungs than did virus rPR8-Mex NS1/230 (Table 1).

**Table 1:** A validation of microarray data by qRT-PCR.

Gene	Microarray fold change(fold change>2.0, q-value <0.05)	qRT-PCR fold change
CXCL1	2.02	5.73
CXCL2	3.47	16.42
CXCL5	2.86	14.27
CXCL9	2.45	4.64
CCL7	2.13	3.24
CCL8	2.28	5.43
IFN-γ	2.63	7.6
IL1β	3.14	10.15
Clec4e	3.75	7.48
Clec4d	2.81	12.95
Clec5a	2.16	4.87
Stfa2l1	4.07	26.6

### The NS1 C-terminal 11-aa truncation did not alter intracellular localization in transfected or infected cells

**Figure 2:**
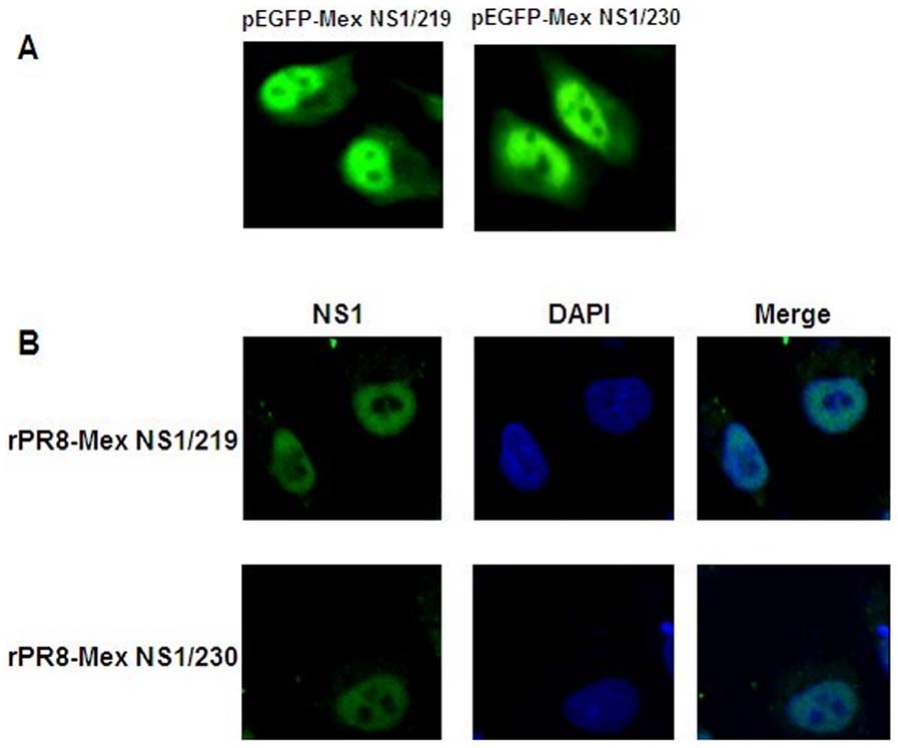
Intracellular localization of different-length NS1 protein in transfected or infected cells. (A) A549 cells were transfected with the plasmid pEGFP-Mex NS1/219 or pEGFP-Mex NS1/230. At 24 h post-transfection, cells were imaged using an inverted fluorescence microscope. (B) A549 cells were infected with recombinant virus rPR8-Mex NS1/219 or rPR8-Mex NS1/230 at an MOI of 3 TCID_50_ per cell. At 8 h p.i., cells were fixed with 4% paraformaldehyde, permeabilized with 0.5% Triton X-100 and incubated for one hour with 2% bovine serum albumin. An NS1-specific rabbit anti-serum incubation was performed overnight at 4°C followed by FITC-labelled goat anti-rabbit IgG and DAPI. Cells were imaged on an LSM510 Meta confocal laser scanning microscope.

Certain critical amino acids (219K, 220R, 224R and 229K) at the C-terminus of NS1 constitute an NoLS [18]. To determine whether the inefficient blocking of host gene expression by the C-terminal 11-aa truncated NS1 of the 2009 pandemic virus was associated with the loss of NoLS, intracellular localization of different-length NS1 from the 2009 pandemic virus in transfected and infected A549 cells was analysed. As shown in Figure 2, both of the 219 and 230 amino-acid length NS1 localized in the nucleus and cytoplasm, but not nucleolus. These results demonstrated that the inefficient blocking of host gene expression by the C-terminal 11-aa truncated NS1 of the 2009 pandemic virus was not associated with nucleolar localization.

### C-terminal truncated NS1 from the 2009 pandemic virus lost PABII-binding capacity

**Figure 3:**
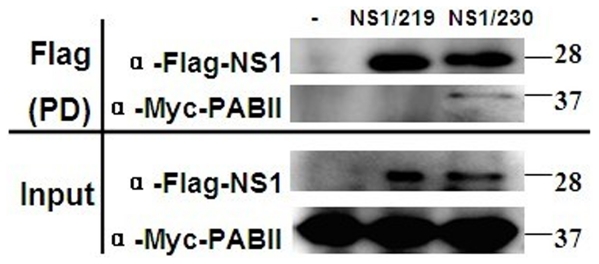
Interactions of different-length NS1 protein with PABII. 293T cells were co-transfected with the MYC-tagged vector pMYC-PABII and the FLAG-tagged vector pCMV-tag2B (empty vector), pCMV-Mex NS1/219 or pCMV-Mex NS1/230. At 48 h post-transfection, cells were lysed, supplemented with a protease inhibitor cocktail and subjected to anti-FLAG affinity chromatography. After extensive washing, the precipitated proteins were dissociated from the resin using elution buffer and analyzed by SDS-PAGE followed by Western bloting with anti-FLAG and anti-MYC antibodies. The proteins were visualized using a SuperSignal West Pico and detected with a MF-ChemiBIS system (DNR). PD: Pull Down.

PABII is involved in host pre-mRNA processing and nuclear export of host mRNA. The C-terminal 223-230 aa of influenza virus NS1 constitute PABII-binding domain [5], and the binding of NS1 to PABII results in inhibition of host pre-mRNA maturation. To determine whether the C-terminal 11-aa truncated NS1 of the 2009 pandemic virus lost the PABII-binding capacity, co-precipitations of FLAG-tagged NS1/219 or NS1/230 with MYC-tagged PABII were performed. The results showed that FLAG-NS1/219 did not coprecipitate with PABII, while FLAG-NS1/230 clearly bound to PABII (Figure 3). Analysis of whole-cell lysates confirmed similar expression levels of the input proteins (Figure 3). These data demonstrated that the inefficient inhibition of host gene expression for NS1/219 was probably due to the loss of PABII-binding capacity.

### *In vitro* characterization of the recombinant viruses with different-length NS1 protein

**Figure 4:**
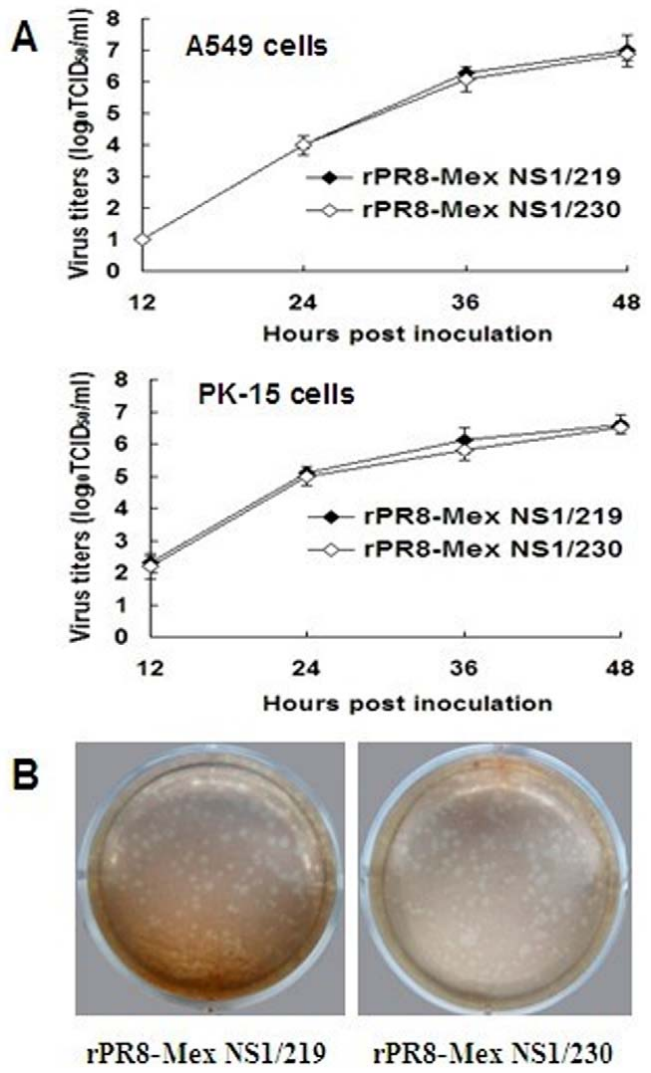
In vitro characterization of the recombinant viruses encoding different-length NS1 protein. (A) A549 or PK-15 cells were infected at an MOI of 0.001 TCID_50_ per cell. After washed with PBS, fresh medium containing 0.1 µg/ml TPCK-trypsin was added. Supernatants were sampled at 12, 24, 36 and 48 h p.i. and virus titers were determined as log_10_TCID_50_/ml in MDCK cells. The statistical analysis was performed using Student's t test. (B) Virus plaque assay was performed by inoculating confluent MDCK cells in 6-well plates with appropriate dilutions of viruses. After washed with PBS, cells were overlaid with DMEM (without phenol red)-0.8% agarose mixture containing 1% bovine serum albumin and 1 µg/ml TPCK-trypsin. After incubated at 37°C for 4 days, a second agar overlay containing 1∶10,000 neutral red was added.

To determine whether the C-terminal 11-aa truncation in NS1 of the 2009 pandemic virus altered virus replication *in vitro*, the growth property in A549 and PK-15 cells and plaque-forming capacity in MDCK cells of the recombinant viruses rPR8-Mex NS1/219 and rPR8-Mex NS1/230 were determined. As shown in Figure 4, The two recombinant viruses exhibited similar growth kinetics in A549 and PK-15 cells (Figure 4A), and formed similar-size plaques in MDCK cells (Figure 4B).

### The NS1 C-terminal 11-aa truncation enhanced virus pathogenicity in mice

**Figure 5:**
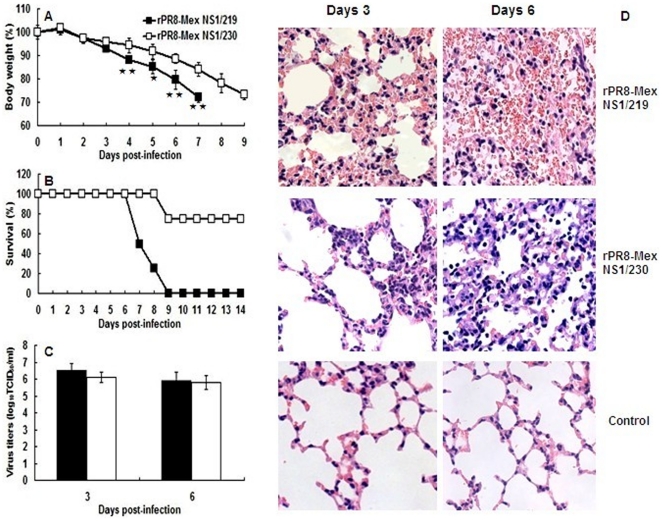
Pathogenicity and growth property of the recombinant viruses encoding different-length NS1 protein in mice. Groups of mice were inoculated i.n. with 50 µl of 10^2^ TCID_50_ of the recombinant virus rPR8-Mex NS1/219 or rPR8-Mex NS1/230. (A) Body weight of five mice in each group was measured for either 14 days p.i. or until the loss of 30% of their body weight. The statistical analysis was performed using Student's t test (★★, *p*<0.01; ★, *p*<0.05). (B) The survival percentage of four mice in each group was monitored for 14 days p.i.. (C) At days 3 and 6 p.i., three mice in each group were sacrificed, lungs were removed and homogenized in 1 ml PBS to determine virus titers by log_10_TCID_50_/ml in MDCK cells. (D) At days 3 and 6 p.i., three mice in each group were sacrificed and lungs were fixed with formalin, embedded in paraffin and stained with hematoxylin and eosin. Images were captured at 40X magnification.

MLD_50_ assays were performed by inoculating groups of four mice with serial 10-fold dilutions of the recombinant virus rPR8-Mex NS1/219 or rPR8-Mex NS1/230. Virus rPR8-Mex NS1/219 displayed slightly lower MLD_50_ titer than virus rPR8-Mex NS1/230 (1.5 or 1.9 log_10_TCID_50,_ respectively). To determine the morbidity (measured by body weight loss) and mortality, groups of mice were infected i.n. with 10^2^ TCID_50_ of virus rPR8-Mex NS1/219 or rPR8-Mex NS1/230. The data showed that virus rPR8-Mex NS1/219 caused more rapid body weight loss and resulted in death earlier than did virus rPR8-Mex NS1/230 (Figure 5A and 5B). Histological analysis of infected mouse lung tissues showed that virus rPR8-Mex NS1/219-infected tissues exhibited more severe hemorrhage and alveolitis than did tissues infected with virus rPR8-Mex NS1/230 (Figure 5D). When growth property of virus rPR8-Mex NS1/219 and rPR8-Mex NS1/230 was assessed in mouse lungs, virus rPR8-Mex NS1/219 grew to slightly, but not significantly higher titers than virus rPR8-Mex NS1/230 at days 3 and 6 p.i. (Figure 5C).

## Discussion

The NS1 protein of influenza A viruses has attracted much attention because of its multifunctional role. Recent reports have showed that the 2009 pandemic virus induced higher amounts of pro-inflammatory cytokines and chemokines in mammalian models than human seasonal H1N1 influenza viruses [3]. In this study, we focused on the C-terminal 11-aa truncation in NS1 of the 2009 pandemic virus. Renilla luciferase expression was used to mimic host gene expression [20], and the results showed that the C-terminal 11-aa truncated NS1 of the 2009 pandemic virus was inefficient at blocking host gene expression, while extension of the truncated NS1 to its full length increased the inhibition of host gene expression. This characterization was further confirmed in A549 cells and mice lungs infected with recombinant virus rPR8-Mex NS1/219 or rPR8-Mex NS1/230. The recombinant virus rPR8-Mex NS1/219 induced higher amounts of IFN-β mRNA production in A549 cells and higher levels of pro-inflammatory cytokines and chemokines in mice lungs than did virus rPR8-Mex NS1/230 (Table 1).

The C-terminal 219-230 aa of H3N2 influenza virus NS1 constitute an NoLS as the four critical amino acids are 219K, 220R, 224R and 229K [18]. Whereas, this is not a case for the H1N1 influenza virus A/WSN/33, of which the four critical amino acids are 219K, 220R, 224G and 229E. Single mutation G224R or double mutations G224R and E229K in NS1 of virus A/WSN/33 led to partial or full nucleolar localization [18]. Extension of the 219-aa NS1 of the 2009 pandemic virus to its full length resulted in the four critical amino acids being 219K, 220R, 224R and 229E. Interestingly, the full-length NS1 did not show partial nucleolar localization in transfected or infected cells. Hale et al. [24] recently identified a new key residue 221K involved in nucleolar localization of NS1 protein. Although the residue 221K was observed in both MSN NS1 and Mex NS1/230, the failure of nucleolar localization for Mex NS1/230 might be due to other unidentified key residues, which were involved in nucleolar localization and different between WSN and Mex NS1.

PABII is involved in cellular pre-mRNA processing, binding of influenza virus NS1 to PABII results in the nuclear accumulation of cellular pre-mRNAs containing short poly(A) tail [9]. The C-terminal 223-230 aa of influenza virus NS1 constitute PABII-binding domain [5]. Here, we determined that the C-terminal 11-aa truncation of NS1 from the 2009 pandemic virus resulted in the loss of NS1's PABII-binding capacity, extension of the truncated NS1 to its full length restored its PABII-binding capacity. Thus, the increased inhibition of host gene expression of 230-aa NS1 might be due to the restoration of its PABII-binding capacity.

It has been reported that the four C-terminal residures of NS1 protein in most human and animal influenza viruses formed an X-S/T-X-V type PDZ ligand motif [25], which has been reported to be associated with virus virulence [26]–[29]. Extension of the 219-aa NS1 of the 2009 pandemic virus to 230-aa resulted in an NS1 protein encoding a GTEI motif at aa 227-230, which is not a PDZ ligand motif. However, the effects of the C-terminal 11-aa truncation of the NS1 protein from the 2009 pandemic virus on virus replication and pathogenicity have attracted much attention recently. Hale et al. [23] indicated that the C-terminal 11-aa truncation in NS1 did not alter the replication or virulence of the 2009 pandemic virus, authors [23] speculated that the biological effects of the C-terminal 11-aa truncation in NS1 would be more apparent if different virus strains or host species were used, as the genetic constellation of the 2009 pandemic virus might already be optimized to function efficiently. In this study, the laboratory strain PR8 was chosen as backbone to generate two “7+1” recombinant viruses expressing either 219-aa or 230-aa NS1 from the 2009 pandemic virus. Our studies showed that the C-terminal 11-aa truncation in NS1 enhanced virus pathogenicity in mice, which was consistent with the reports of Ozawa et al. that the C-terminal 11-aa truncation in NS1 of the 2009 pandemic virus increased virus virulence in mice [30].

In summary, we determined that the C-terminal-truncated NS1 of the 2009 pandemic virus was inefficient at blocking host gene expression, extension of the truncated NS1 to its full length increased the inhibition of host gene expression, restored its binding capacity to PABII, but decreased virus pathogenicity in mice.
